# Evaluation of dental implant with hydroxyapatite coating by laser-induced hydrothermal synthesis: in vitro and in vivo experimental study

**DOI:** 10.1186/s11671-025-04330-7

**Published:** 2025-08-12

**Authors:** Jung-Tae Lee, Sungtae Kim, Sung-Ho Lee, Dong-Wook Han, Keonhee Lee, Sangyun Lee, Daehyeok Kwon, Kyungwoo Lee, Hojeong Jeon, Tae-Gon Jung, Bongju Kim

**Affiliations:** 1 Department of Periodontics, One-Stop Specialty Center, Seoul National University Dental Hospital, Seoul, Republic of Korea; 2 Department of Periodontology, Dental Research Institute, Seoul National University School of Dentistry, Seoul, Republic of Korea; 3 Dental Life Science Research Institute, Seoul National University Dental Hospital, Seoul, Republic of Korea; 4 Department of Cogno-Mechatronics Engineering, Pusan National University, Busan, Republic of Korea; 5 Dental Implant Research Center, OSSTEO Bionics INC, Incheon, Republic of Korea; 6 Biomaterials Research Center, Biomedical Research Division, Korea Institute of Science and Technology, Seoul, Republic of Korea; 7 Medical Device Development Center, Osong Medical Innovation Foundation, Chungju, Republic of Korea

**Keywords:** Titanium, Dental implants, Surface modification, Osseointegration

## Abstract

Various surface modification techniques have been developed to improve the survival rate of dental implants. This study aimed to evaluate both in vitro and in vivo outcomes of implants coated with a nano/micro-assembled hydroxyapatite (HA) structure using a laser-induced single-step coating (LISSC) technique. Four types of implant surfaces were examined: machined surface implants (MA), sandblasted large-grit acid-etched implants (SLA), resorbable blasting media implants (RBM), and HA-coated implants (HA). In vitro analyses included surface morphology, surface hydrophilicity, and cell attachment. Twelve rabbits and two beagle dogs were used in the in vivo experiments. The implant stability quotient (ISQ) was measured immediately after placement and again at sacrifice (rabbits: 3 and 6 weeks; beagles: 12 weeks), followed by histological evaluation and quantification of bone-to-implant contact (BIC%) and bone volume (BV%). ISQ values increased from the postoperative period to 6 or 12 weeks across all implant types. In vitro, surface roughness ranked as HA > RBM > SLA > MA, while surface wettability ranked as RBM > HA > MA > SLA. No significant differences were observed in initial cell adhesion or viability among the groups. In vivo, BV ranked as MA > RBM > SLA > HA at 3 weeks, and MA > HA > RBM > SLA at 6 weeks. BIC ranked as RBM > MA > SLA > HA at 3 weeks and HA > RBM > SLA > MA at 6 weeks. HA exhibited the greatest increases in both BV and BIC from 3 to 6 weeks. In beagles, ISQ at 12 weeks was higher than baseline for both SLA and HA, with HA demonstrating superior BV compared to SLA. Within the limitations of this preclinical study, HA-coated implants produced via the LISSC method demonstrated comparable or superior biological performance relative to conventional MA, SLA, and RBM surfaces.

## Introduction

Dental implants are a widely accepted treatment modality for replacing missing teeth and improving quality of life [1]. Two critical determinants of implant success are the timing of placement and the achievement of osseointegration [2]. Patient discomfort, both functional and esthetic, tends to increase with a prolonged edentulous period. To address this, early or immediate loading protocols have been introduced [3]. Osseointegration—the direct structural and functional connection between living bone and the implant surface—is essential for long-term success [4]. This process involves continuous load transfer within bone tissue without intervening connective tissue [5].

Osseointegration occurs through both distance and contact osteogenesis [6]. Factors influencing this process include implant biocompatibility, design, surface characteristics, host bone quality, surgical technique, and loading conditions. When these conditions are suboptimal, inflammatory tissue may develop between the implant surface and alveolar bone, resulting in implant failure [7]. Clinically, osseointegration can be assessed using resonance frequency analysis (RFA) or the periotest method [8]. Yeo et al. reported that rough implant surfaces promote both contact and distant osteogenesis [9]. Primary stability is therefore a prerequisite for successful osseointegration and is strongly influenced by surface modifications. Accordingly, various strategies have been developed to alter implant surface topography and chemistry, with the goal of enhancing primary stability and reducing healing time [10].

Surface properties such as topography, wettability, and coating significantly influence osteoblast function and bone formation [11]. Recent advances include nanotopography modification, anodic oxidation, ultraviolet (UV) photofunctionalization, and antibacterial or micropatterned coatings [10]. Previous studies have demonstrated that nanoscale surface features enhance the initial biological response of osteoblasts and osteoclasts [12]. A recent meta-analysis showed that short implants with nanostructured calcium-incorporated titanium surfaces, hydrophilic sandblasted acid-etched surfaces, or dual acid-etched surfaces coated with nanometer-scale calcium phosphate crystals had reduced marginal bone loss compared with longer implants [2]. This highlights the potential of surface modification to overcome the inherent disadvantages of short implants. Various molecules, such as polycations, bactericidal materials, and physical vapor deposition (PVD) [13].

Among coating materials, hydroxyapatite (HA) has been widely used due to its biocompatibility, bioactivity, and osteoconductivity [14]. As the principal mineral component of bone and teeth, HA offers slow biodegradation, space maintenance, delayed resorption, and enhanced stability during early bone remodeling [15]. Doping HA with antibacterial ions such as silver or strontium further enhances its antimicrobial properties [16]. However, conventional HA coatings produced by plasma spraying or physical vapor deposition (PVD) often suffer from poor adhesion, thermal damage, and unpredictable resorption [17].

There have been various attempts to create nanostructured layers, including anodization and three-dimensional (3D)-printed porous titanium (pTi) alloys [18, 19]. Despite the wide clinical adoption of HA coatings for implant osseointegration, conventional methods such as plasma spraying and PVD are limited by poor coating adhesion, high thermal damage, and unpredictable resorption rates. To address these limitations, recently, a method for making a “nano/micro-assembly structure of hydroxyapatite” using a laser called “laser-induced single-step coating (LISSC)” has been introduced. LISSC was newly developed by complementing the existing HA coating method using physical and chemical methods [20]. The LISSC process is as follows; substrates (Titanium: Ti or magnesium: Mg) in a supersaturated calcium and phosphate(CaP) solution are treated with a nanosecond pulsed laser ->HA penetrates into the molten substrate by the laser, forming a strong bond. Through in vitro experiments, Um et al. proposed that HA-coated Mg using a nanosecond laser showed improved adhesion strength, resulting in increased resistance to corrosion and biocompatibility [21]. However, limited in vivo data are available on LISSC-coated implants, particularly in comparison with other commercially available surface modifications.

Therefore, this study aimed to evaluate the results of in vitro and in vivo experiments on implants using a recently developed nano/micro-assembled structure of the hydroxyapatite’ method in comparison with SLA, RBM and MA implants. In vivo experiments were conducted in both rabbits and beagles to minimize interspecies variability and enhance translational relevance.

## Methods

### In vitro experiment: surface characterization

#### Specimen Preparation

A Ti6Al4V (Grade 5) specimen, used as a substrate for surface modification, was prepared in the form of a bar with flat surfaces on both sides. The size of the specimen is 2 mm x 8 mm. (TNS Korea, Incheon, Korea)

MA group: It was made with without any additional surface treatment after machining.

SLA group: It was prepared by sandblasting (3 bar 6 s) with alumina 0.05 μm range powder (Al_2_O_3_) followed by etching with an acid solution (hydrogen chloride: HCl 30% + sulfuric acid: H_2_SO_4_ 35%). The process is carried out for 8 min at a temperature of 75 to 80 degrees.

RBM group: It was fabricated by blasting (Asung Sanding, Gyeonggi-do, Korea) with CaP based powder. Blasting is a suction tube type blasting of powder by compressed air. In this way, the diameter of the nozzle is 1 mm and the distance from the specimen is 8 cm, The surface that is blasted using four nozzles is the entire specimen I made it possible. The amount of powder used in blasting is blasting 3 kg/cm2 with a constant 400 mg/min regardless of pressure Blasting under blasting pressure for 12 s to blast the titanium surface RBM-treated uniformly.

HA group: It was performed on the coating solution using a 1064 nm wavelength nano-second ytterbium fiber laser (Biolino MOPA, Laservall, Hong Kong). The Ti surface was irradiated over a 3 × 7 mm area under laser conditions of 23 W pulse power, 550 mm/s scan speed, 500 kHz repetition rate, and 500 ns pulse duration, and loop number of 70. After surface treatment, it was cleaned using an ultrasonic cleaner in deionized water for 10 min, six times.

Dulbecco’s modified Eagle medium (DMEM, LM001-09, Welgene, Seoul, Korea) was used as a basic precursor to prepare the HA sample coating solution. To increase the concentration of Ca and P ions, two aqueous solutions, a 1 M H_3_PO_4_ solution and a 2 M CaCl_2_ solution, were prepared by diluting calcium chloride (C5670, Sigma-Aldrich, Oakville, ON, Canada) and 85 wt% phosphoric acid (345245, Sigma-Aldrich) in deionized water. The Ti samples were immersed in the DMEM solution containing the calcium chloride and phosphoric acid then irradiated with a laser.

#### Analysis of surface characterization

Surface morphology analysis was performed using a scanning electron microscope (SEM) (Inspect F50, FEI, USA). Before SEM analysis, all samples were coated with Pt for 60 s at a current of 15 mA. SEM analysis was conducted using a secondary electron (SE) detector. Images were obtained at an accelerating voltage of 15 kV with magnifications of 30x, 2,000x, and 30,000x.

Surface elemental analysis was conducted using energy-dispersive X-ray spectroscopy (EDS) (Inspect F50, FEI, USA). EDS mapping was performed on images obtained at a magnification of 2,000x using SEM, with the surface elements Ca, P, and Ti mapped in green, purple, and mint, respectively.

Surface roughness was measured with a 3D laser scanning microscope (OLS5000, Olympus, Tokyo). The surface was scanned at 100x magnification to obtain 3D images, followed by surface roughness analysis (*n* = 3).

Surface hydrophilicity was assessed using contact angle analysis (SmartDrop, Femtofab, Korea). A water droplet of approximately 10 µL was placed on the sample surface, and the contact angle was measured after 10 s (*n* = 3).

### In vitro experiment: cell attachment

#### Sample Preparation and cell seeding

Samples were affixed to the bottom of 48-well plates using medical adhesive. Subsequently, 1.0 × 10^3^ cells/sample of mouse-derived osteoblasts (MC3T3-E1, ATCC, Rockville, USA) were seeded in droplet form onto the flat surface of the samples, which were coated using various techniques. The cells were suspended in 20 µL of minimum essential medium (α-MEM without phenol red, Gibco, Massachusetts, USA) containing 10% fetal bovine serum (FBS, HyClone, Logan, USA) and 1% antibiotic-antimycotic (P/S 100x, HyClone). The samples were incubated for 30 min at 37 °C in a humidified atmosphere with 5% CO_2_ to allow for initial cell adhesion.

**Fig. 1:**
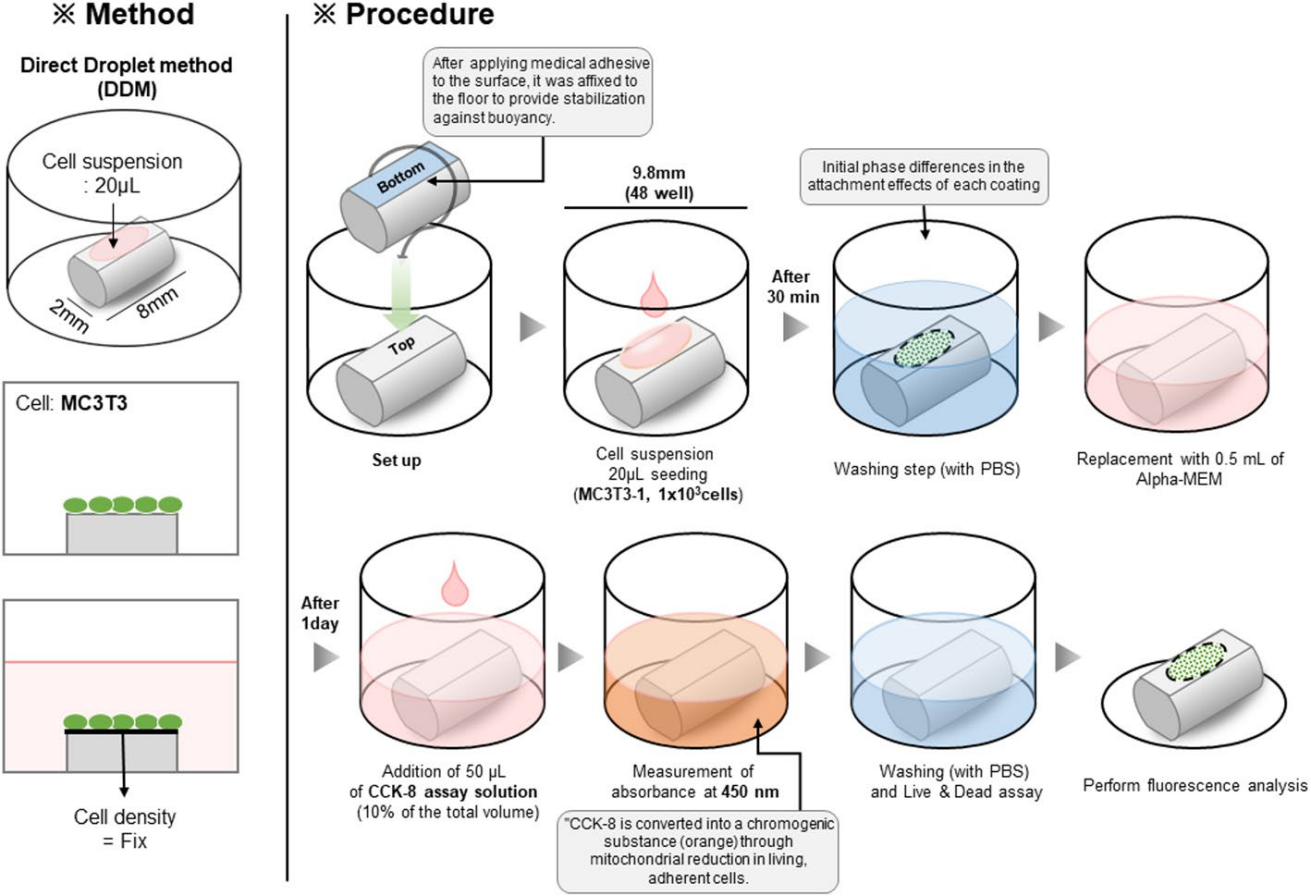
The direct droplet method (DDM) was used to seed MC3T3-E1 osteoblasts onto samples affixed to the bottom of 48-well plates with medical adhesive. A cell suspension (1.0 × 10^3^ cells in 20 µL α-MEM with 10% FBS and 1% P/S) was applied, followed by a 30-minute incubation. Non-adherent cells were removed by washing with PBS, the medium was replenished, and the samples were incubated for an additional 24 h. Cell viability and proliferation were assessed using a CCK-8 assay, where 50 µL of reagent was added, followed by a 2-hour incubation and absorbance measurement at 450 nm. Additionally, a live/dead assay was performed using Ethidium Homodimer-1 and Calcein AM staining, with cell attachment visualized via fluorescence microscopy

After the 30-minute incubation, non-adherent cells were gently washed away with phosphate-buffered saline (PBS, Welgene). The culture medium was then replenished with 500 µL of fresh medium, and the samples were incubated for an additional 24 h under the same conditions (Fig. 1).

#### Cell viability and proliferation assays

To evaluate cell viability and proliferation, a CCK-8 assay was performed according to the manufacturer’s instructions (Dojindo, Kumamoto, Japan). After 24 h of incubation, 50 µL of CCK-8 reagent (10% v/v of total culture media volume) was added to each well, and the plates were incubated for an additional 2 h at 37 °C in a 5% CO_2_ humidified incubator. Absorbance at 450 nm was measured using a microplate reader (Fig. 1).

#### Live/dead cell assay

For visual assessment of cell attachment, a live/dead assay was conducted. The culture medium was replaced with serum-free medium containing Ethidium Homodimer-1 and Calcein AM (R37601, Thermo Fisher Scientific, OH, USA). The stained cells were then observed using a fluorescence microscope (Axio Imager.A2m, Carl Zeiss, Oberkochen, Germany) (Fig. 1).

### In vivo experiment: rabbit and beagle

#### Preparation of animals

Animal experiments were approved by the Institutional Animal Care and Use Committee (IACUC) of Seoul National University (rabbit: IACUC No. SNU-221012-1; beagle: No. SNU-221012-4). The experimental animals were male New Zealand white rabbits (2–3 kg; KNOTUS, Incheon, Korea) and male beagle dogs (~ 15 kg; KNOTUS). The care and use of animals and surgical procedures were performed in accordance with the Korean Guide for the Use and Care of Laboratory Animals and IACUC regulations.

#### Preparation of specimen

In this study, machined surface implants (MA), sandblasted, large grit, acid-etched surface implants (SLA), resorbable blasting media surface implants (RBM), and hydroxyapatite surface implants (HA) were used. The four implant groups were obtained from OSSTEO Bionics (Gimpo-si, Korea).

SLA implant - SB implant (OSSTEO Bionics, Gimpo-si, Korea): The surface of the fixture is sandblasted with alumina (Al_2_O_3_) powder on the surface of MA implant, and then the surface is treated with SLA (Sandblasting with large grit and acid etched) coating instrument (SAND BLAST M/C, Asung Sanding).

RBM implant - SB implant (OSSTEO Bionics): RBM modification treatment was performed by blasting with CaP-based powder on the MA surface. The implants were fabricated under the same conditions as those used in the in vitro experiments.

HA implant - SB implant (OSSTEO Bionics): The equipment used for coating was a nanosecond (ns) ytterbium fiber laser (JPT 30w, SK Laser, korea) with a wavelength of 1064 nm. The laser powers 30 W were used in this process. After one side of MA implant was coated, the coating was rotated 45 degrees using a rotating stage, a total of 4 times. After coating, cleaning was carried out using 6 ultrasonic waves (UIL-1 H-1200w, uilultrasonic co. ltd, Ansan-si, Korea).

#### Surgical procedures

The sample size was estimated based on previous studies by Roy TR et al. (rabbit) and Kuki Y et al. (beagle) [22–24], which reported significant differences in bone-implant contact with comparable protocols. Using G*Power software version 3.1 [25], a one-way ANOVA was assumed with an effect size (f) of 0.4, α = 0.05, and power (1–β) = 0.90, resulting in a required minimum sample size of 6 implants per group for the rabbit model. The beagle experiment was conducted as a preliminary pilot study to assess feasibility and trends prior to larger-scale studies.

#### Rabbit

MA group [*N* = 12 (3 weeks; *n* = 6 and 6 weeks; *n* = 6)]: 3.5 Ø × 6 mm, SB implant (OSSTEO Bionics).

SLA group [*N* = 12 (3 weeks, *n* = 6; and 6 weeks, *n* = 6)]: 3.5 Ø × 6 mm SB implant (OSSTEO Bionics).

RBM group [*N* = 12 (3 weeks; *n* = 6 and 6 weeks; *n* = 6)]: 3.5 Ø × 6 mm, SB implant (OSSTEO Bionics).

HA group [*N* = 24 (3 weeks; *n* = 12 and 6 weeks; *n* = 12)]: 3.5 Ø × 6 mm, SB implant (OSSTEO Bionics).

**Fig. 2:**
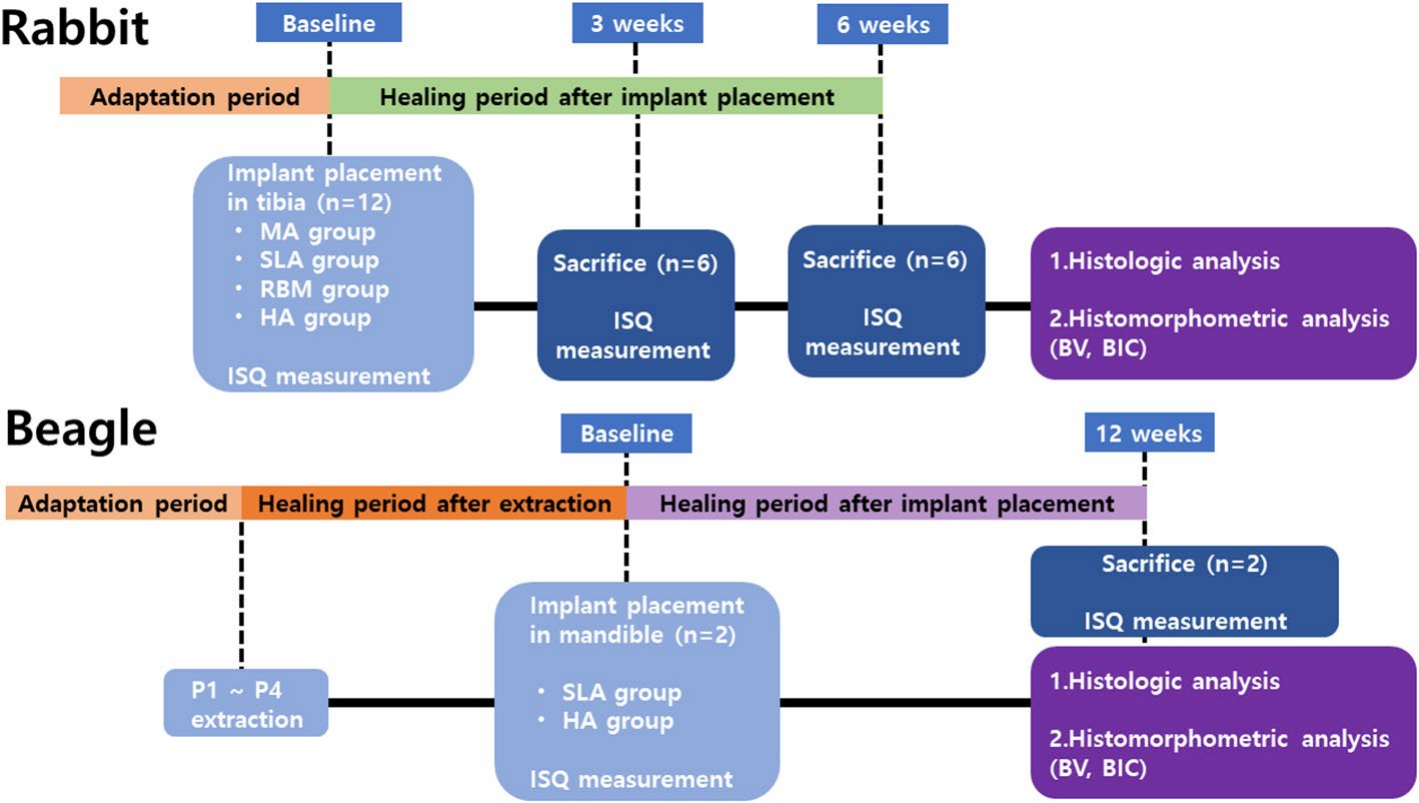
Flow diagram of the study. MA, machined surface implants; SLA, sandblasted, large grit, acid-etched surface implants; RBM, resorbable blasting media surface implants; HA, hydroxyapatite surface implants; ISQ, implant stability quotient; BV, bone volume ratio; BIC, bone-implant contact ratio

**Fig. 3:**
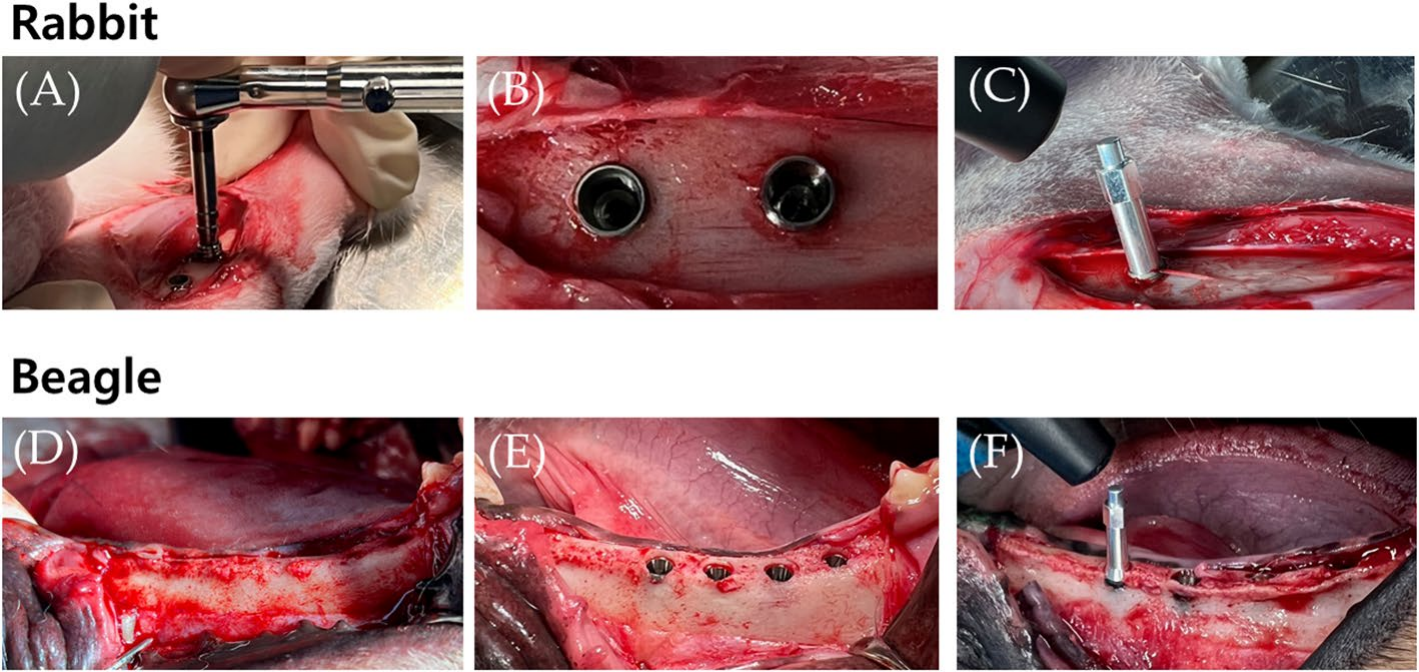
Surgical procedure of rabbit and beagle **A**–**F**. Implant placement **A**, **B**, **D**, and **F**. ISQ measurement with transducer **C** and **F** ISQ, implant stability quotient

Twelve rabbits were used for the in vivo experiment. A mixture of xylazine hydrochloride (Rompun^®^ 5 mg/kg, Bayer, Seoul, Korea) and ketamine hydrochloride (Ketara^®^, 35 mg/kg, Yuhan, Seoul, Korea) was intravenously injected for general anesthesia. All fur on the left and right hind legs was removed, the skin in the surgical area was cleaned with povidone-iodine, and an approximately 2 cm incision was made in the skin. After the removal of the muscles and periosteum, the tibia was exposed. One implant was placed 10 mm inferior to the cancellous bone of the tibial shaft using a low-speed handpiece, and a second implant was placed 15 mm apart. After measuring the implant stability quotient (ISQ) using an Osstell mentor device (Integration Diagnostics AB, Göte-borg, Sweden), the implants were sutured in the reverse order of surgery after tying the cover screw. Three and six weeks after implant placement, an incision was made at the implant placement site to expose the implant for ISQ measurements (Figs. 2 and 3).

#### Beagle

SLA group (*N* = 8): 4.0 Ø × 10 mm, SBA implant (OSSTEO Bionics).

HA group (*N* = 8): 4.0 Ø × 10 mm, SB implant (OSSTEO Bionics).

Beagle dogs: This study was conducted using two beagles. A mixture of xylazine hydrochloride (Rompun^®^ 5 mg/kg, Bayer) and ketamine hydrochloride (Ketara^®^, 35 mg/kg, Yuhan) was administered intravenously for general anesthesia. After extracting premolars (#1–4) from the left and right sides of the mandible, a 3-month recovery period. The gums of the recovered mandibles were incised, and implants were placed in extracted positions. HA implants were placed in the right mandible, and SLA implants were placed in the left mandible. Immediately after implant placement, the ISQ was measured using an Osstell mentor device (Integration Diagnostics AB, Sweden), the cover screw was tightened, and the implant was sutured in the reverse order of surgery. The ISQ was measured 12 weeks after implant placement, and the animals were sacrificed. After each rabbit and beagle dog was sacrificed during the experimental period, the bone at the implant placement site was cut and fixed in 10% paraformaldehyde. The specimens were cut into resin blocks, and slides were prepared and stained with hematoxylin and eosin staining (H&E) (Figs. 2 and 3).

### Histomorphometric analysis

Samples were fixed in a neutral 10% formaldehyde solution for 48 h. They were then washed with water for 3 h and dehydrated with 80–100% alcohol. Samples were placed in Technovit^®^ (Jorgensen Labs, Colorado, USA) 7200 VLC base solution resin, stirred, and embedded for 3–5 days. Acrylic slides with resin blocks attached were cut into 40–50 μm thick sections, mounted on slides, and subjected to H&E staining. Stained slides were subjected to histomorphometric analysis using a light microscope (Nikon Micropho-to-FXA, Nikon Corporation, Tokyo, Japan) equipped with a Kodak digital camera (Kodak Professional DCS 420; Eastman Kodak Company, Rochester, NY, USA). The bone in contact with the implant threads was photographed, and the BIC% and bone volume ratio (BV%) were measured and analyzed.

### Statistical analysis

All data were processed using GraphPad Prism 9.0 (GraphPad Software, Boston, MA, USA) and expressed as the mean ± standard deviation. All data were evaluated using one-way analysis of variance, and Tukey’s test was used for post-hoc tests. Results were considered statistically significant if the P-value was < 0.05.

## Results

### In vitro experiment: surface characterization

**Fig. 4:**
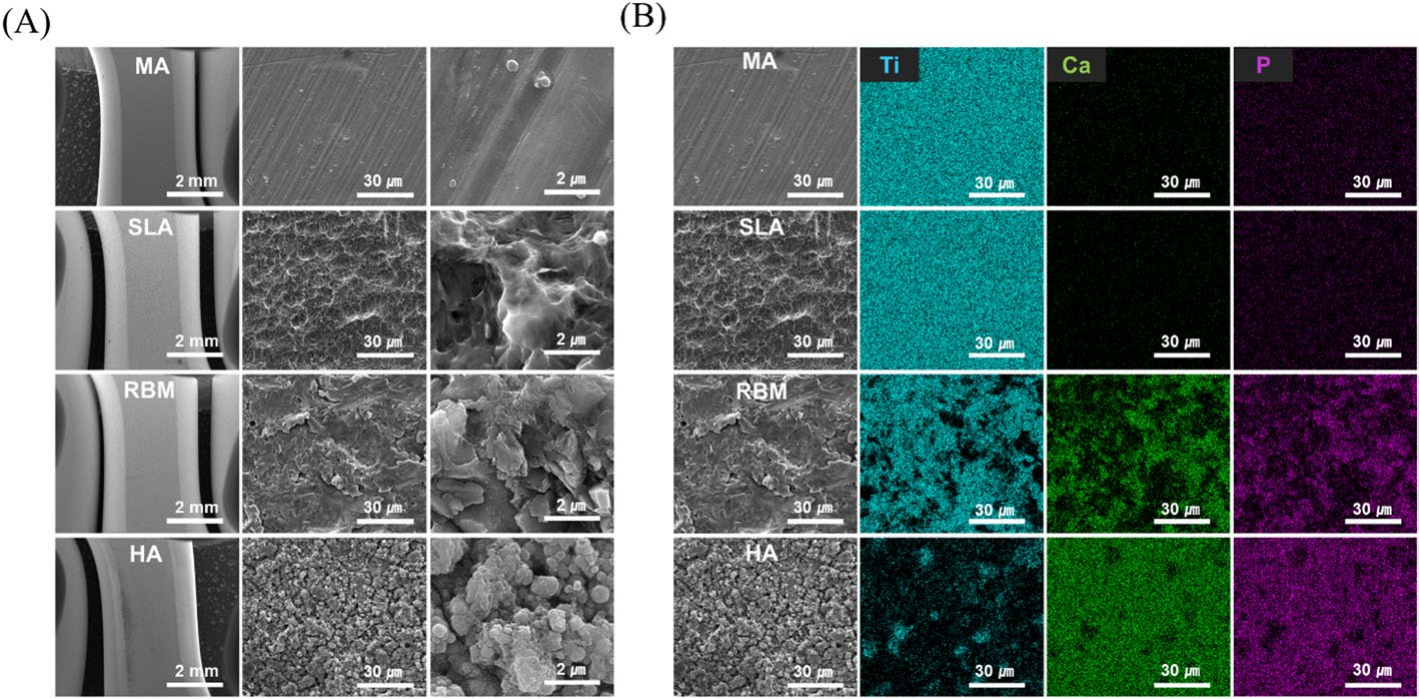
Surface structural and composition characterization according to surface treatment method. **A** Surface morphology analysis using SEM. **B** Surface element analysis via EDS mapping image

To examine the surface morphology, SEM analysis was conducted (Fig. 4A). SEM images revealed clear micro- and nano-topographic differences across the four groups. In particular, the HA group exhibited granular structures of hydroxyapatite particles distributed across the surface, distinguishing it from the smoother machined (MA) surface. The MA group showed periodic grooves patterns due to machining, while the other surface-treated samples exhibited the formation of microstructures on the surface. For the SLA sample, small pits caused by blasting were observed, and the RBM sample displayed a mountain-like structure. In the case of HA, microstructures were formed on the surface after treatment, but unlike the previous conditions, hydroxyapatite powder of several hundred nanometers(nm) in size was observed on the surface. To analyze the elemental composition of the coated surfaces, EDS mapping analysis was performed (Fig. 4B). For the Ti and SLA samples, no ions other than Ti were detected. In contrast, Ca and P ions were detected in the RBM and HA samples. In the case of HA, the ratio of Ca and P was confirmed to be 1.67 as a result of surface component analysis through EDS. In the RBM sample, the presence of Ca and P ions on parts of the surface was attributed to the resorbable bioceramic powder used to create the roughness. For the HA sample, the primary components of hydroxyapatite, Ca and P ions, were evenly distributed across the surface, and the Ti surface was almost completely covered. This confirmed that hydroxyapatite was uniformly coated on the surface.

**Fig. 5:**
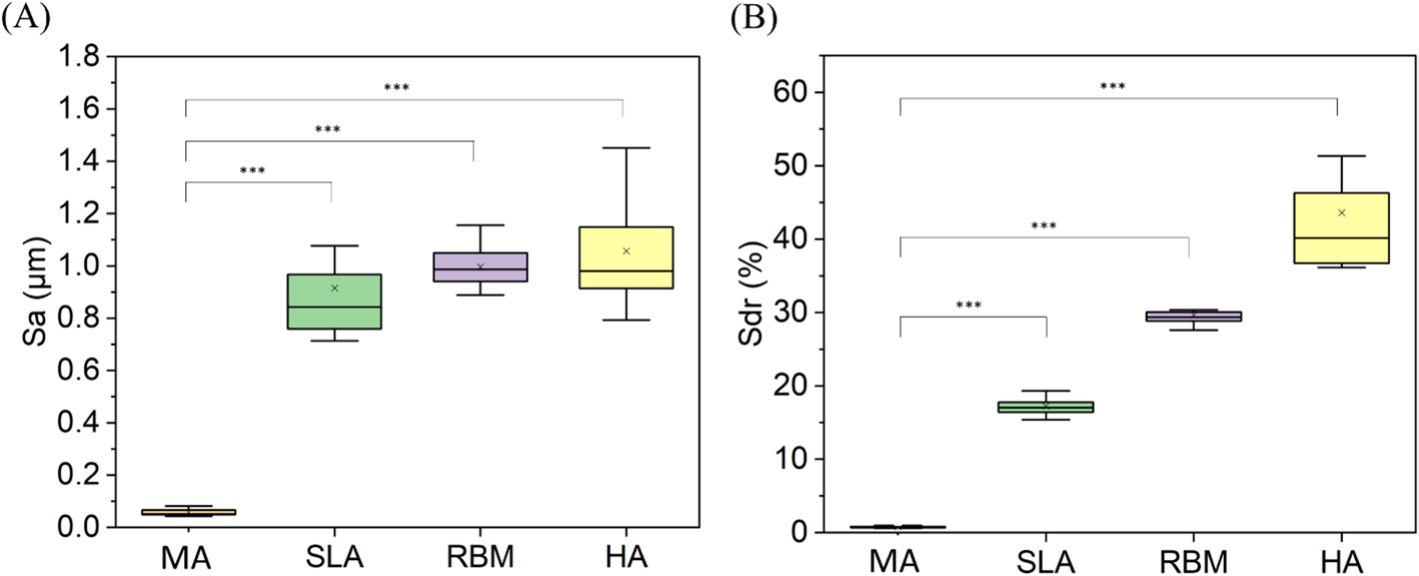
Comparison of surface roughness according to surface treatment method. **A** Arithmetic mean height (Sa) was measured via 3D optical microscope. **B** Surface area ratio (Sdr) was measured via 3D optical microscope. Statistical significance is indicated as follows: *** (*p* < 0.001)

The surface roughness was measured using 3D optical microscopy. Quantitatively, surface roughness (Sa) increased significantly in the HA group (1.06 ± 0.21 μm) compared to MA (0.06 ± 0.01 μm), SLA (0.92 ± 0.21 μm) and RBM (0.99 ± 0.08 μm), with statistical significance (*p* < 0.001). (Fig. 5A).

In addition, the surface area ratio (Sdr) values for the MA, SLA, RBM, and HA samples were 0.79 ± 0.14%, 17.21 ± 1.07%, 29.66 ± 1.62%, and 43.60 ± 7.87%, respectively (Fig. 5B).

**Fig. 6:**
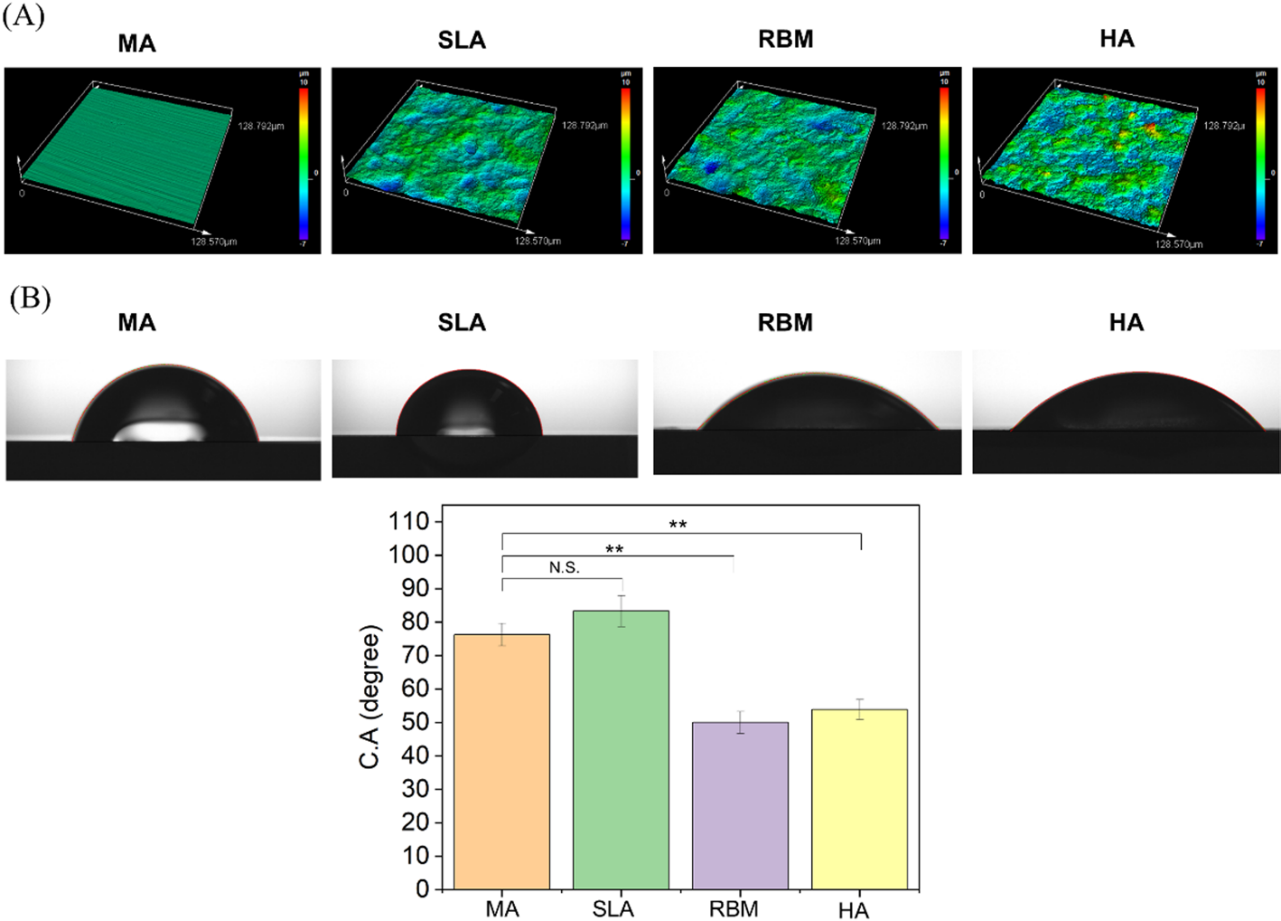
Comparison of surface properties according to surface treatment method. **A** Surface morphology analysis using 3D optical microscope. **B** Surface wettability analysis through contact angle. Statistical significance is indicated as follows: Statistical significance is observed as follows: N.S. (Not Significant), **(*P* < 0.01), *** (*p* < 0.001)

The surface roughness was measured using 3D optical microscopy (Fig. 6A). Observing the images obtained from 3D scanning, it was confirmed that the surface-treated samples had increased roughness compared to the MA sample. Statistical significance is indicated as follows: *** (*p* < 0.001) Significant difference (***, *p* < 0.001) were found in the comparisons of MA vs. SLA, MA vs. RBM, and MA vs. HA.

In Fig. 6B, the contact angle (C.A) values are compared between the MA group and the other experimental groups (SLA, RBM, and HA). All conditions showed contact angles below 90 degrees, indicating hydrophilic properties. While the wettability of the SLA sample did not increase compared to the MA sample, the RBM and HA surfaces showed improved wettability compared to both the MA and SLA samples. This increase in hydrophilicity was attributed to the presence of hydroxyapatite, which has inherently hydrophilic properties. Statistical significance is observed as follows: N.S. (Not Significant), **(*P* < 0.01). The comparison of MA vs. SLA showed no significant differences. Significant differences (**, *P* < 0.01) were found in the comparisons of MA vs. RBM and MA vs. HA.

### In vitro experiment: cell attachment

**Fig. 7:**
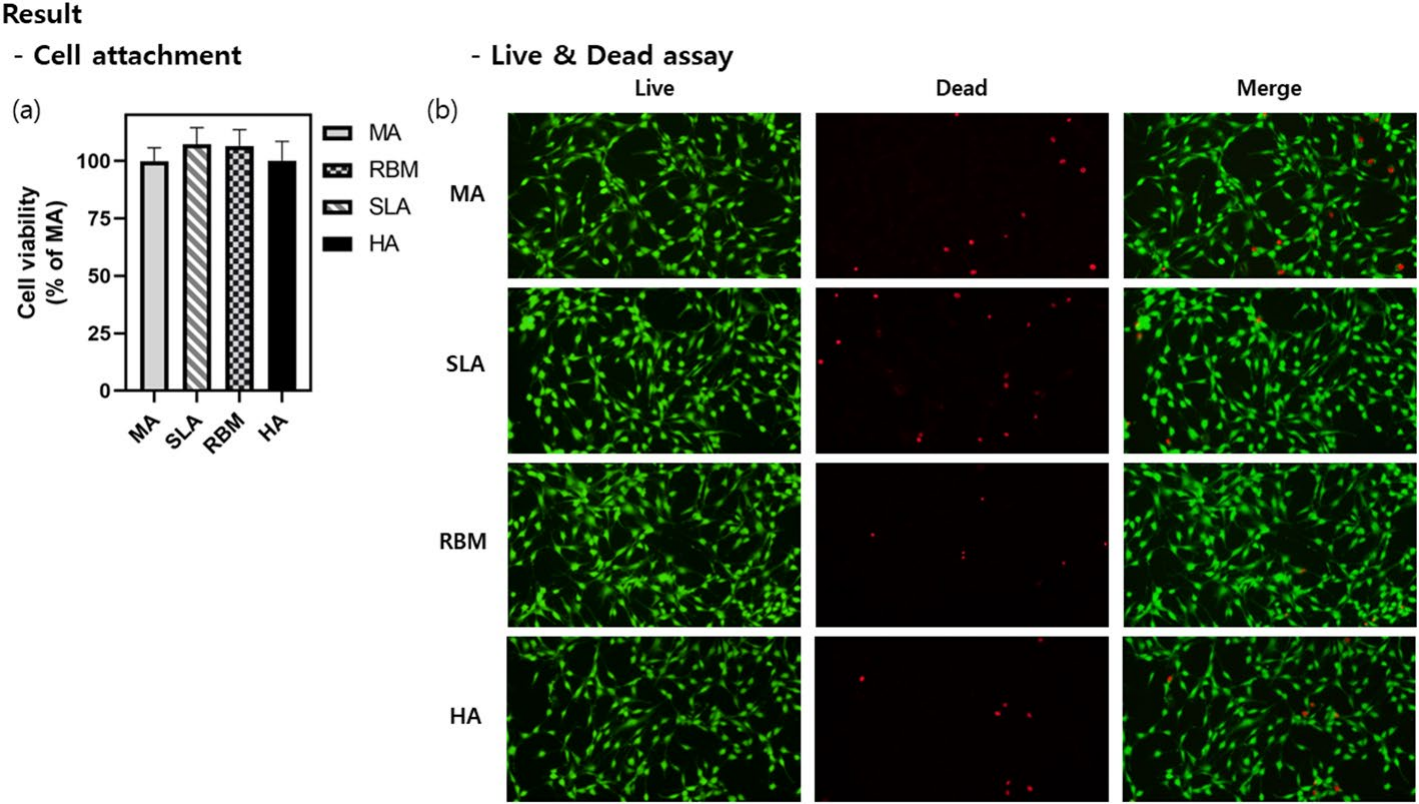
Evaluation of Initial cell adhesion and viability on coated samples. **A** Cell viability—absorbance at 450 nm, indicative of cell viability, after 24 h of incubation. Different coating techniques show significant variation in cell viability. **B** Live/Dead Cell Assay—Fluorescence microscopy images showing live (green) and dead (red) cells on the coated samples, illustrating the effectiveness of various coatings on initial cell adhesion and survival (color figure online)

The initial cell adhesion and viability on samples treated with different coating techniques (MA, RBM, SLA, HA) were evaluated using a series of assays. The CCK-8 assay was performed to assess cell viability after 24 h of incubation. The absorbance measurements at 450 nm, which indicate cell viability, showed no significant differences between the samples with different coatings (Fig. 7A). This suggests that the initial adhesion and subsequent viability of MC3T3-E1 cells were not significantly affected by the surface treatments. Conversely, it indicates that the cells did not exhibit toxicity in response to any of the coated surfaces.

Additionally, the live/dead cell assay provided further confirmation of these results. Fluorescence microscopy images demonstrated a similar distribution of live (green) and dead (red) cells across all samples, irrespective of the coating technique used (Fig. 7B). This indicates that the various surface treatments did not lead to significant variations in initial cell adhesion or viability.

### In vivo experiment: implant stability quotient (ISQ)

#### Rabbit

**Fig. 8:**
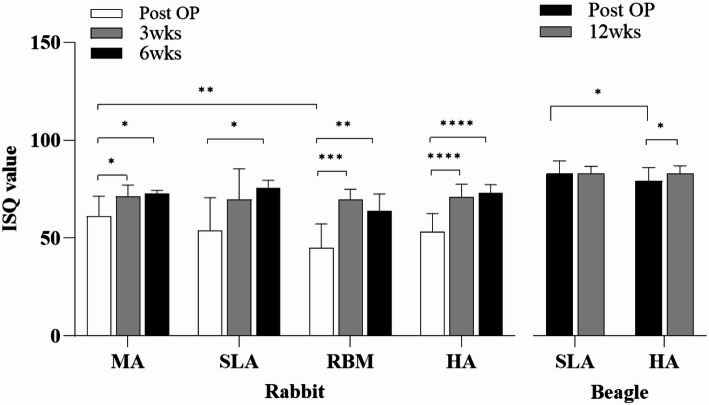
ISQ values for MA, RBM, SLA, and HA implants in the tibia of rabbits. SLA and HA implants in the mandible of beagles. MA: machined surface implants; SLA: sandblasted, large grit, acid-etched surface implants; RBM: resorbable blasting media surface implants; HA: hydroxyapatite surface implants; ISQ: implant stability quotient. * *p* < 0.05, ** *p* < 0.01, *** *p* < 0.001, **** *p* < 0.0001

Figure 8 shows the results of the ISQ analysis. In all groups, ISQ values increased overall for 6 weeks after surgery (post-OP: 61.00 ± 10.00; 3 weeks: 71.30 ± 6.00; 6 weeks: 72.90 ± 1.60). At 6 weeks, the ISQ values were similar across all groups. There were significant differences when comparing the outcomes at post-OP vs. 3 weeks and post-OP vs. 6 weeks (*P* < 0.05). No statistically significant differences were observed at 3 or 6 weeks. The SLA group shows the results as follows: 53.90 ± 17.20 (post-OP), 69.70 ± 17.20 (3 weeks), 75.80 ± 4.10 (6 weeks) Only the value of post-OP vs. 6 weeks was shown as the statistical difference (*P* < 0.05). In the RBM group, the ISQ values of all groups were similar after 6 weeks. The 6-week value was seen to have decreased compared to the 3rd week (post-OP: 44.90 ± 12.60, 3 weeks: 69.60 ± 5.30, 6 weeks: 64.00 ± 9.30). The results of the ISQ analysis for RBM showed a significant interaction between post-OP vs. 3 weeks and post-OP vs. 6 weeks (**P < 0.01, ***P < 0.001). No difference was found in the ISQ value between 3 and 6 weeks. The HA group has similar results to other groups (post-OP: 53.15 ± 9.50, 3 weeks: 71.10 ± 6.50, 6 weeks: 73.00 ± 4.50). A statistical difference is found post-OP vs. 3 weeks and vs. 6 weeks (*P* < 0.0001). There is also significant difference between MA and RBM groups in post-OP.

#### Beagle

The ISQ values in the beagles are also summarized in Fig. 8 [73.88 ± 4.46 (post-OP), 77.38 ± 3.71 (12 weeks)/SLA group: 77.25 ± 6.27 (post-OP), 77.47 ± 3.42 (12 weeks)]. In the HA group, there is a significant difference between post-OP and 12 weeks (* *P* < 0.05). However, no association is observed between post-OP and 12 weeks in the SLA group. A significant difference is shown between SLA and HA in post-OP (* *P* < 0.05).

### In vivo experiment: bone volume (BV)

#### Rabbit

**Fig. 9:**
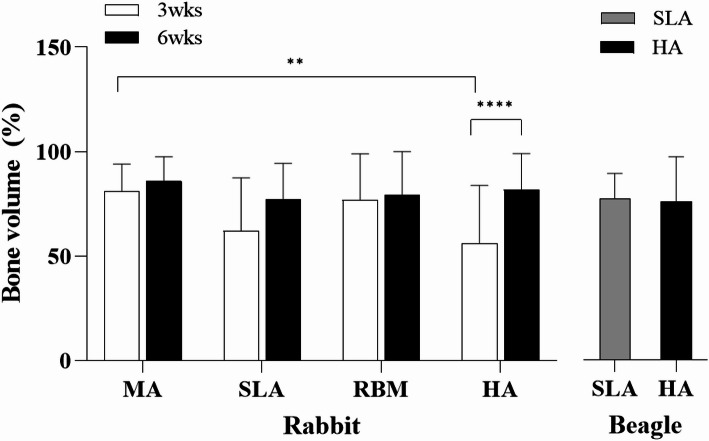
BV values **A**–**D** for MA, RBM, SLA, and HA implants in the tibia of rabbits. **E** SLA and HA implants in the mandible of beagles. MA: machined surface implants; SLA: sandblasted, large grit, acid-etched surface implants; RBM: resorbable blasting media surface implants; HA: hydroxyapatite surface implants; ISQ: implant stability quotient; BV: bone volume ratio. ** *p* < 0.01, **** *p* < 0.0001

Figure 9 shows the BV% of MA, SLA, RBM, and HA in rabbit tibia. At week 3, HA is lower than MA (9.31%), SLA (30.91%), and RBM (26.90%). At week 6, HA was 5.90% higher, SLA is 4.74% lower, and RBM was 3.21% higher than MA. Compared with week 3, the values of all groups increase by 24.76% (MA), 5.66% (SLA), 3.19% (RBM), and 45.68% (HA) at week 6. There are significant differences when comparing the outcomes at MA vs. HA groups at 3 weeks (*P* < 0.01) and 3 weeks vs. 6 weeks in HA group (*P* < 0.0001).

#### Beagle

The changes in bone volume in beagles are also shown in Fig. 9. The BIC of the HA implants was approximately 1.7% higher than that of the SLA implants. No statistically significant differences were observed between the groups.

### In vivo experiment: bone implant contact (BIC: %)

#### Rabbit

**Fig. 10:**
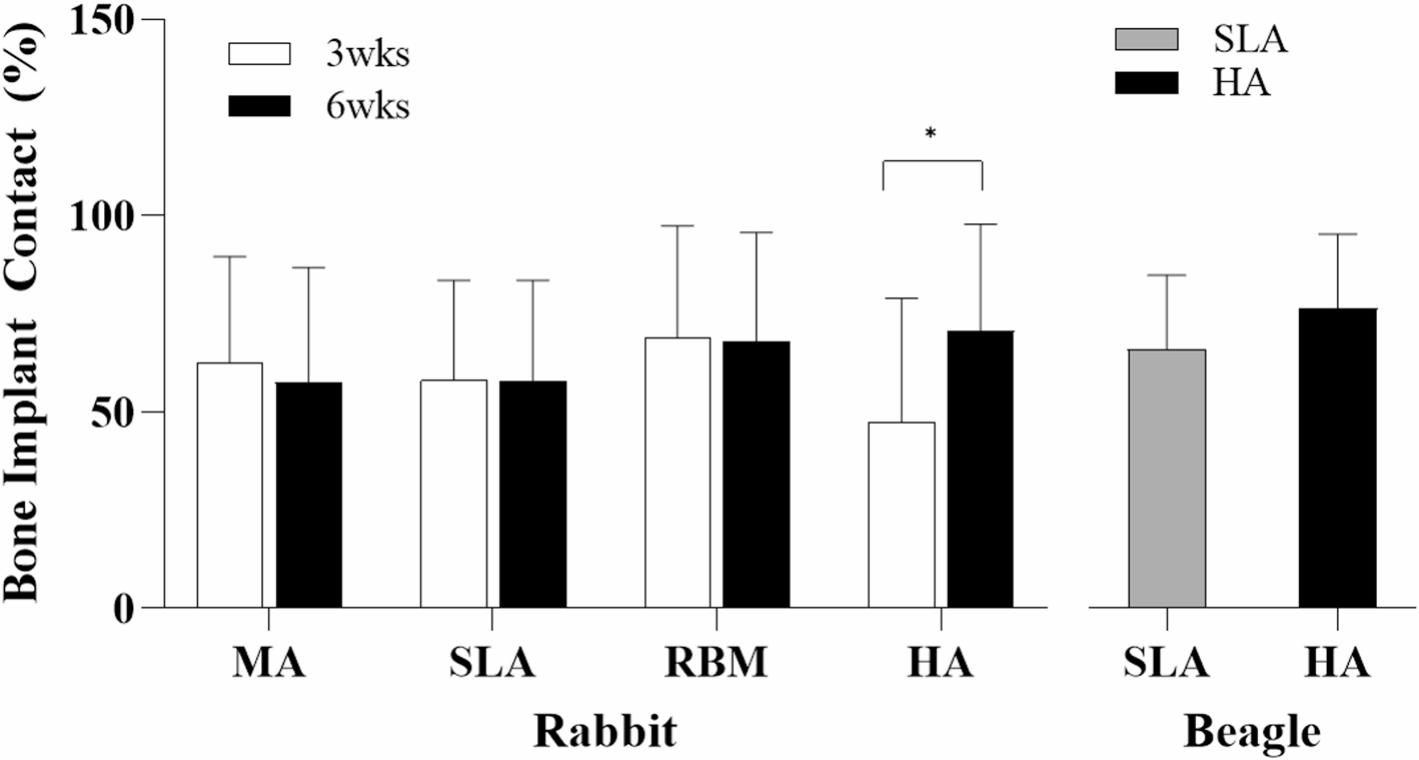
BIC values **A**–**D** for MA, RBM, SLA, and HA implants in the tibia of rabbits.(**E** SLA and HA implants in the mandible of beagles. MA, machined surface implants; SLA, sandblasted, large grit, acid-etched surface implants; RBM, resorbable blasting media surface implants; HA, hydroxyapatite surface implants; BIC, bone-implant contact ratio * *p* < 0.05

The changes in bone A BIC in all groups were shown, respectively (MA, 62.4%; SLA, 58.1%; RBM, 68.8%; and HA, 65.8%) (Fig. 10). In the HA group, at 3 weeks, the BIC values were lower than those in the MA (24.19%), SLA (18.53%), and RBM (31.19%) groups. However, the HA value at 6 weeks was higher than that of the other groups (MA: 2.02%, SLA: 22.37%, and RBM: 3.86%). Compared with the 3rd week, in the 6th week, MA was 7.91% lower, SLA was 0.51% lower, RBM was 0.99% lower, and HA was 49.43% higher. A statistical difference is observed 3 vs. 6 weeks in HA group.

#### Beagle

The BIC of the HA implants was approximately 15.86% higher than the SLA implants. No statistically significant differences were observed between the groups (Fig. 10).

### In vivo experiment: histologic analysis

#### Rabbit

Week 3.

**Fig. 11:**
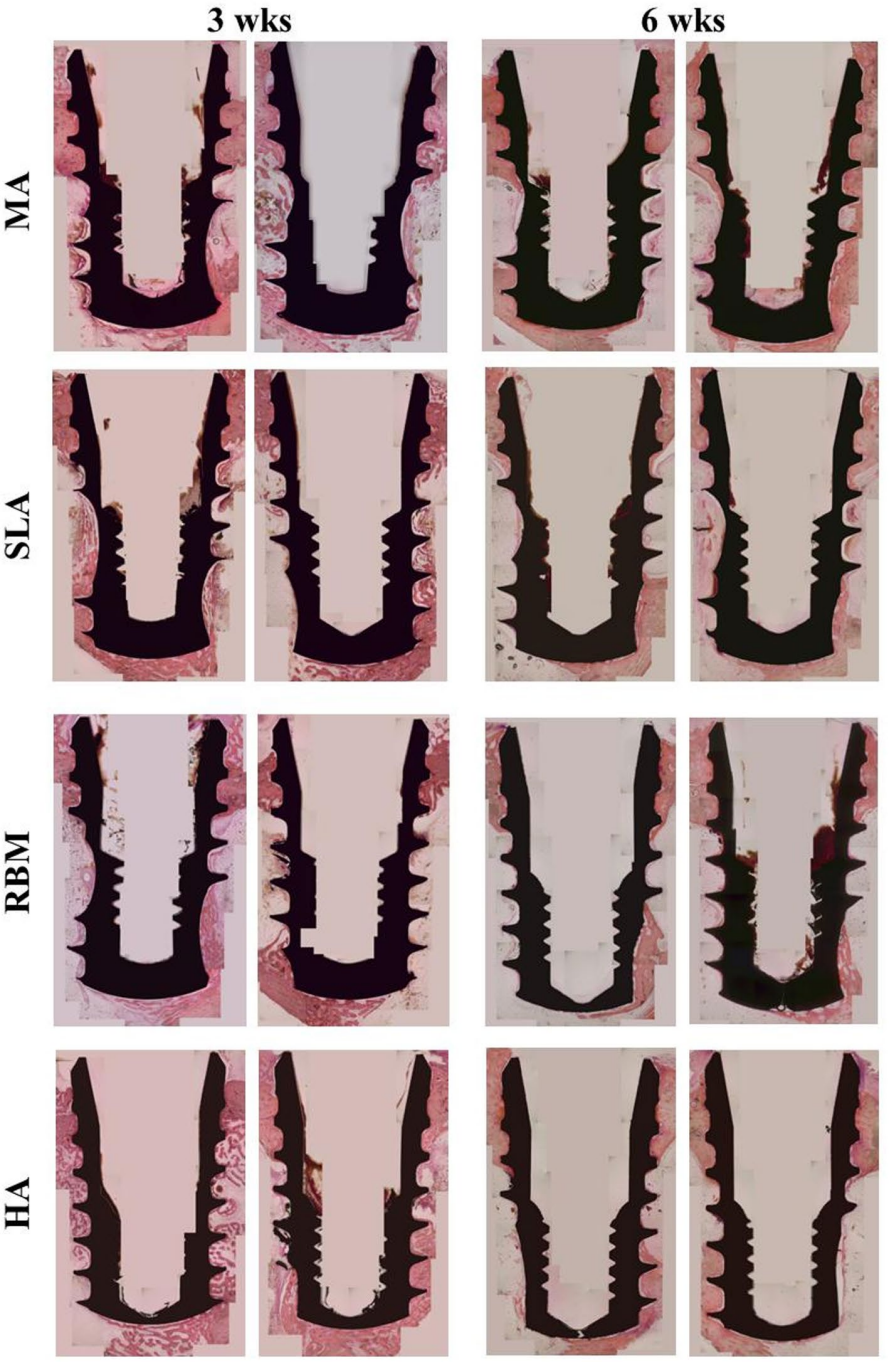
Histological image (hematoxyline & eosin x20) in the tibia of rabbits.MA: machined surface implants; SLA: sandblasted, large grit, acid-etched surface implants; RBM: resorbable blasting media surface implants; HA: hydroxyapatite surface implants

No fractures occurred during implant placement in any group. However, adverse inflammatory reactions have been observed in bone marrow in several cases. Histological findings in the HA group were similar to those in the MA, SLA, and RBM groups. All groups showed osteoid and mineralized bones between the implant surface and the cortical border in the tibia. Newly formed trabecular bone was observed on the implant surface in all groups. Fibrous tissue was observed at the implant apex (Fig. 11).

Week 6.

No inflammation was observed in any implant group. Newly formed mature bone was observed between the coronal part of the implant and the tibial cortical border. Histological analyses revealed a higher quantity of new mature bone surrounding the implant body and apex (Fig. 11).

#### Beagle

Week 12.

**Fig. 12:**
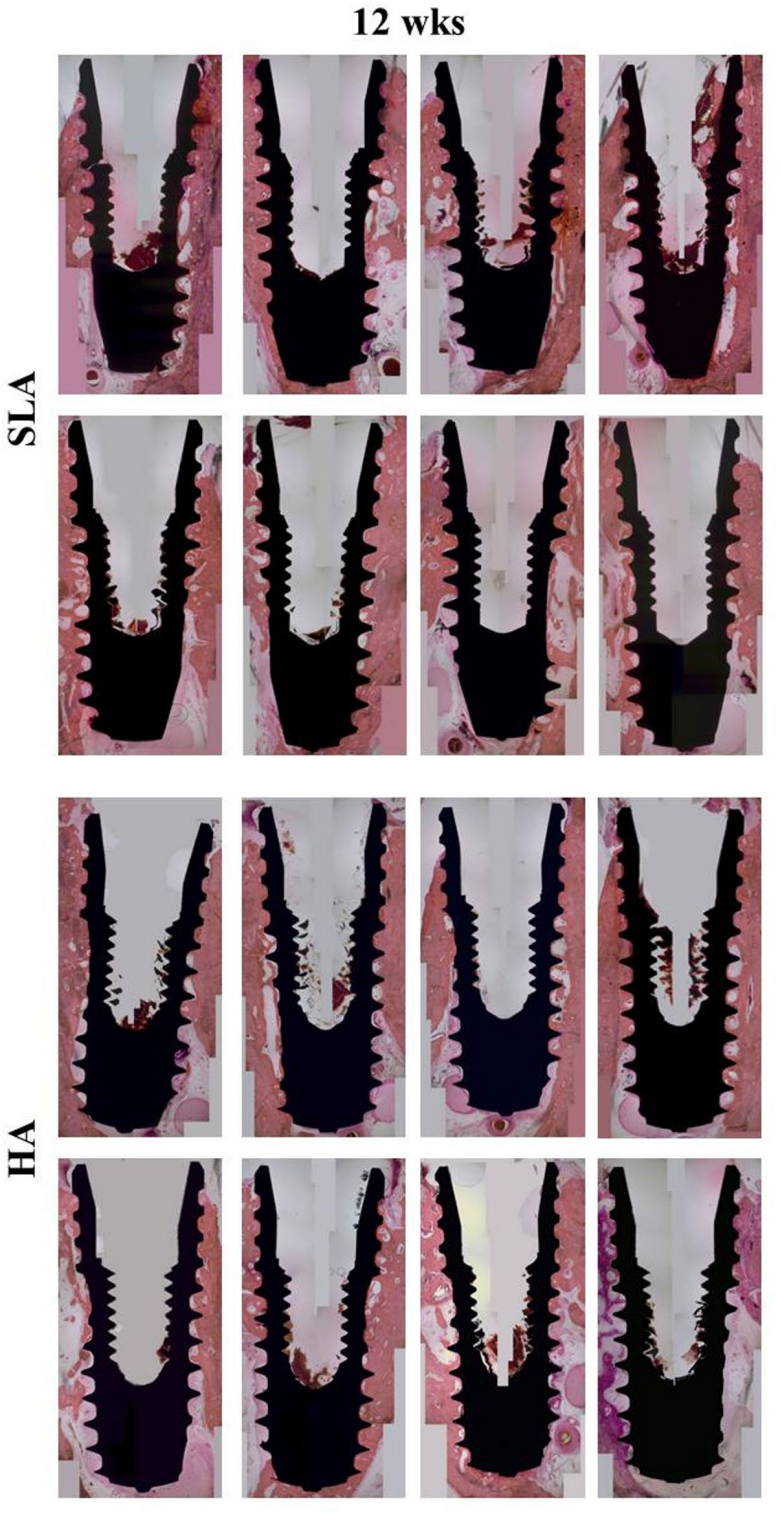
Histological image (hematoxyline & eosin x20) in the mandible of beagles. SLA: sand-blasted, large-grit, acid-etched surface implants; HA: hydroxyapatite surface implants

No adverse inflammatory events were observed in either group. The healing patterns of the two types of implants were similar. New bone formation was observed tightly surrounding the HA and SLA implant surfaces. Vascular canals and partial remodeling of bone trabeculae were observed in the newly formed bone areas. Osteocyte lacunae were also observed in trabecular bones (Fig. 12).

## Discussion

This study evaluated the in vitro and in vivo performance of implants coated with a recently developed nano/micro-assembled hydroxyapatite (HA) structure using the LISSC technique, in comparison with implants treated with conventional surface modifications (MA, SLA, RBM). In vitro analyses demonstrated that surface roughness ranked as HA > RBM > SLA > MA (Sa and Sdr values), while wettability ranked as RBM > HA > MA > SLA. No statistically significant differences in initial cell adhesion or viability were observed among the groups, suggesting that the coating method does not critically influence the earliest stages of MC3T3-E1 osteoblast attachment. In vivo, ISQ values progressively increased across all groups, indicating stable osseointegration (> 50 by Osstell Mentor^®^). HA showed the greatest increase in BV between 3 and 6 weeks, while BIC values ranked as RBM > MA > SLA > HA at 3 weeks and HA > RBM > SLA > MA at 6 weeks. Notably, HA exhibited the largest gains in both BV (45.7%) and BIC (49.4%) between 3 and 6 weeks, supporting previous reports that nanoscale topography promotes osteoblast activity and mineral deposition [11, 12]. These results are consistent with Bingul et al. who reported enhanced osseointegration in rat models following local application of zoledronic acid, further emphasizing the role of surface characteristics in modulating early bone response [26]. Histological findings at 3 weeks revealed inflammatory reactions and localized bone loss in the marrow cavity, which may explain the variability in early BV and BIC measurements. By 6 weeks, however, inflammation was absent, and new bone formation was evident, particularly in the HA group. In beagles, both HA and SLA demonstrated higher ISQ values at 12 weeks compared with baseline, while BIC was higher for HA than SLA, with no significant differences in BV between the two groups.

Resonance frequency analysis (RFA) proved to be a reliable indicator of implant stability, as reported previously [27–29]. In this study, ISQ values were consistent across groups, though occasional very low postoperative readings were observed, likely due to technical factors such as poor transducer contact or microfractures in the rabbit tibia [30]. Interestingly, HA implants showed a gradual and consistent increase in ISQ values, aligning with prior reports of delayed but progressive stability in HA-coated implants [31, 32].

BIC remains one of the most reliable histomorphometric parameters for assessing osseointegration, with values above 50% generally considered indicative of stable bone–implant integration [33, 34]. In this study, all implant types exceeded 50% BIC, consistent with prior findings [35]. The implant surface is moderately rough and has hydrophobicity with a contact angle of 90 degrees or more. This property lowers the wettability of blood and is disadvantageous for the adhesion of cells to serum [36]. To overcome this problem, many studies have been conducted on implant surface modifications. Contact angle analyses showed that all groups had hydrophilic surfaces (< 90°), with HA and RBM exhibiting significantly greater wettability than SLA and MA, providing a more favorable environment for cell adhesion. Previous reports similarly demonstrated long-term clinical success of SLA implants [37] and superior BIC for RBM surfaces [38]. However, the contact angle values of RBM and HA groups were significantly smaller than those of other SLA and MA groups. This can be considered that the environment for cell adhesion may be more favorable in the HA and RBM groups.

Recent research has shown that there are more than sixty methods of biocompatible calcium orthophosphate (CaPO_4_) deposits. Hong et al. found that comparing four different implant surfaces, the average BIC ratio was 95.4% (*P* < 0.01) in the HA coating group, 87.1% (*P* < 0.05) in the RBM group, and 86.0% (*P* < 0.05) in the SLA group [39]. Despite its clinical popularity, conventional HA coating has several drawbacks, including brittleness, poor adhesion, and unpredictable resorption in inflammatory or acidic environments [40–42]. In a previous study, HA coating resorption was reported in an acidic environment caused by inflammation at the osteotomy site. Physical HA coatings also risk thermal damage to the substrate, while chemical nucleation-based methods are complex and time-consuming.

LISSC was introduced as an alternative to overcome the shortcomings of physical and chemical HA coating methods. LISSC employs the effect of hydrothermal synthesis on a laser-irradiated substrate (Mg or Ti) in the liquid (coating precursor solution) interface. Using LISSC, HA particles can be synthesized with a substrate within seconds. with hydroxyapatite particles ranging from nanometer to micrometer. The photoheat generated by the laser on the substrate in the liquid precursor improves the adhesion of the HA coating through precipitation and growth of HA, and increases the corrosion resistance and cell adhesion of the substrate. In addition, various coating layers can be formed depending on the substate and solution used [20, 21]. In previous studies that used Mg as the substate, Mg(OH)_2_ is formed through a natural reaction in an aqueous solution, and Mg(OH)_2_ can supply OH- that can form the HA surface. When the formation of Mg(OH)_2_ is promoted through the laser, more HA coating can be formed on the Mg surface [43, 44]. Considering results of in vitro and in vivo experiments in this study, through the LISSC process, nanoparticle ions (HA, Ca and P) may lead to increase surface wettability, surface energy, attachment of serum proteins. It seems to affect bone formation on the implant surface.

In a previous study, the laser-induced coating layer allows CaP coating adhesion to be achieved with a minimum thickness, which leads to an improvement in the overall corrosion resistance of the biodegradable metal. As a result of a peel test between the thermally formed HA region (TFH) and the laser-induced HA (LIH) coating region, the adhesive strength of the LIH region increased by about 26.52% compared to the TFH region [21]. This formed a strong bond between the substrate and HA, enabling rapid restoration of mechanical defects in the coating layer [45, 46]. However, LISSC requires accurate laser pulse and concentration of coating precursor solution. Park et al. have shown that when the concentration of the solution increases, a large amount of hydrogen gas is generated on the surface of the substrate, which leads to dispersion of the laser beam, and that float occurs during the laser processing process, leading to scattering of the beam [21]. Another study reported that surface coating does not occur with too weak laser pulses (5 and 10w) during the LISSC process. It was also observed that a solution which is a combination of NaF and HMTA was required for coating to be formed [20].

In this study, HA showed similar results when compared to other three groups. The important factor is the long-term follow up of the surface treatment method, which is one of the causes of peri-implantitis. Because LISSC forms a mixed layer by melting the surface of the substrate with a laser, it seems to reduce the possibility of detachment for medium using simple bonding method. The BIC value of HA group (rabbit) increased sharply from 3 to 6 weeks, and in that of HA group (beagle), HA showed a higher value than SLA. This can be considered that the HA layer formed by the LISSC method influenced bone healing. In addition, LISSC is considered to be helpful for the long-term prognosis of implants. In this study, The Sa value was similar to the SLA, RBM, and HA values ​​except for MA, but the Sdr value of HA showed a significant difference compared to other three groups. This indicates that the HA group has a relatively wide surface area as well as peaks and valleys compared to RBM and SLA. The greater Sdr value of the HA group suggests a more complex topography, which increases the available surface area for protein adsorption and initial cell anchorage—both essential for initiating bone remodeling [15]. This is also considered to be helpful for the long-term prognosis of implants.

When processing Ti6Al4V titanium alloy using LISSC, it was observed in XRD and TEM tests that the crystallinity of HA was low. This is considered to be because as HA becomes nanoparticles, the surface energy increases, and as a result, more serum proteins are attached, and bone conductivity also increases [47]. In addition, when the HA layering amount was high, the number of serum proteins and osteocytes attached to the surface increased. Thickness of HA layer seems to increase surface roughness, and the specific area increases accordingly [21].

This study has some limitations. First, this study lacked a sample size and other groups in the beagle dog study. Second, only preclinical studies were conducted. Third, only experiment of initial attachment of cells was conducted. Therefore, further studies should aim to include clinical evaluations of HA and other dental implants and large sample size assessments. In addition, in vitro experiments are needed to assess the long-term changes in cell adhesion, but also to evaluate the expression of bone regeneration–related messenger ribonucleic acid (mRNA) and proteins, including bone morphogenetic protein-2 (BMP-2), runt-related transcription factor 2 (RUNX2), alkaline phosphatase (ALP), osteocalcin (OCN), and osteopontin (OPN). Based on the successful preclinical study results, it is thought that future clinical studies and long-term follow-up studies can be conducted.

## Conclusion

Although the HA group initially showed lower ISQ, BIC, and BV values, these parameters increased substantially over time, with HA surpassing SLA and RBM at later stages. Within the limitations of this preclinical study, implants coated with HA using the LISSC technique demonstrated comparable or superior biological performance to conventional surfaces. These findings suggest that LISSC may represent a promising strategy for enhancing early osseointegration and long-term implant stability.

## Data Availability

All data generated or analyzed during this study are included in this published article.
